# The Role of TLR2/TLR4 Receptors in Host Genetic Susceptibility to Recurrent Vulvovaginitis

**DOI:** 10.3390/jof11110804

**Published:** 2025-11-12

**Authors:** John Routsias, Chrysoula Verra, Aristotelis Tsiakalos, Athanasios Tsakris, Maria Mavrouli

**Affiliations:** Department of Microbiology, Medical School, National and Kapodestrian University of Athens, 75 Mikras Asias, 11527 Athens, Greece

**Keywords:** RVVC, vulvovaginal candidiasis, TLR2, TLR4, Toll like receptors, polymorphisms

## Abstract

Vulvovaginal candidiasis (VVC) is a prevalent vaginal infection predominantly attributed to *Candida albicans*. A considerable proportion of women experience more than three episodes of VVC annually, a condition referred to as recurrent vulvovaginal candidiasis (RVVC). It is estimated that RVVC affects more than 130 million women globally each year and has a substantial negative impact on their quality of life, resulting in physical discomfort, psychological distress, and social stigma. Nevertheless, not all individuals who develop VVC progress to RVVC, suggesting that genetic variation may play a critical role in host susceptibility. The present review aims to evaluate the associations between genetic predispositions—specifically polymorphisms in Toll-like receptors 2 and 4 (TLR2, TLR4)—and RVVC. TLRs are essential for detecting pathogen-associated molecular patterns (PAMPs) and initiating immune responses. During RVVC episodes, *Candida* undergoes a reversible transition from the yeast form to the hyphal form, resulting in alterations in surface PAMPs, which are subsequently recognized by innate immune receptors expressed on vaginal epithelial cells. Polymorphisms in these receptors may modulate individual susceptibility to RVVC. This review examines the literature on the impact of specific polymorphisms in TLR2 and TLR4 on fungal recognition and infection. Furthermore, the interactions between TLRs and other elements of the innate immune system have also been explored. A deeper understanding of how genetic variability in immune receptors influences infection susceptibility could pave the way for personalized therapeutic strategies for RVVC, potentially involving immunomodulatory agents or antifungal treatments tailored to an individual’s genetic profile.

## 1. Introduction

Vulvovaginal candidiasis (VVC) is a fungal infection of the tissue of the vagina and vulva of the female genital tract that is caused mainly by *C. albicans*, an opportunistic fungal pathogen. Over 90% of VVC cases are attributed to *C. albicans*, whereas the remaining 10% are caused by nonalbicans *Candida* (NAC) species, such as Nakaseomyces glabratus (formerly *C. glabrata*), Pichia kudriavzevii (formerly *C. krusei*), *C. tropicalis* and *C. parapsilosis* [1,2,3]. Recurrent vulvovaginal candidiasis (RVVC) is characterized by three or more symptomatic episodes of vulvovaginal candidiasis (VVC) within a year. RVVC is estimated to affect more than 130 million women in any given year, with a global annual prevalence of 3871 per 100,000 females [4]. Although most women report experiencing RVVC for 1–2 years, some women endure recurrent infections for 4–5 years or even for decades [5] This condition significantly impacts the quality of life of affected women, causing physical discomfort, psychological distress, and social stigma [6,7]. In qualitative research interviews, women with RVVC stated high levels of anxiety and concern about social contacts and dating, as well as aversion to sexual engagement [8]. RVVC also has significant economic costs [8]. In the United States, the total yearly insurer and out-of-pocket expenditures for outpatient VVC treatment were estimated to be $368 million in 2017. Furthermore, the projected yearly economic burden of RVVC on the United States in 2010, owing to lost work hours, was $1 billion [4,9,10].

The pathogenesis of RVVC is multifactorial and involves environmental factors, genetic predispositions and immune responses, including the role of Toll-like receptors (TLRs) in the innate immune response [11,12].

## 2. Methodology of the Literature Search

This review included primary studies retrieved from the “PUBMED” database and Google Scholar. The search was conducted during the first half of 2025 using the following keywords: “Recurrent vulvovaginal candidiasis”, “RVVC”, “pathogenesis”, “innate immunity”, “TLR2”, “TLR4”, “Asp299Gly”, “rs4986790”, “Thr399Ile”, “rs4986791”, “Arg677Trp”, “rs121917864”, “Arg753Gln” “rs5743708”. Only English language papers were included. No time limit was placed on the type of published studies. Articles were selected based on their relevance to the topic and the quality of the evidence presented.

## 3. Innate Immunity and Toll-like Receptors in RVVC

### 3.1. Innate Immunity and Toll-like Receptors

Toll-like receptors, as evolutionarily conserved pattern recognition receptors, play a pivotal role in identifying pathogen-associated molecular patterns (PAMPs) [13,14], damage-associated molecular patterns (DAMPs) [15] and initiating immune responses. These transmembrane proteins trigger signaling cascades that lead to the production of proinflammatory cytokines, which are essential for orchestrating the body’s defense against infections [15,16]. TLRs are classified into several types, each recognizing specific PAMPs. Thus, TLR2 recognizes bacterial lipopeptides from Gram-positive bacteria, TLR4 detects lipopolysaccharides (LPSs) from Gram-negative bacteria, and TLR3 is activated by double-stranded RNA [17,18] from viruses. This specificity enables the innate immune system to tailor its response to various pathogens effectively. The engagement of TLRs with their ligands triggers intracellular signaling pathways, primarily the MyD88-dependent and TRIF-dependent pathways, which culminate in the activation of transcription factors such as nuclear factor kappa B (NF-κB) and interferon regulatory factors (IRFs), leading to the expression of genes involved in inflammation and immune responses [18].

During vulvovaginal candidiasis infection, PAMPs on the *Candida*’s surface are recognized by the innate immune system receptors, which activate intracellular signaling within vaginal epithelial cells [19]. These signals stimulate a proinflammatory cytokine response that recruits immune cells, such as phagocytes and T cells, to eradicate the fungus [20].

### 3.2. Ligands of TLR2 and TLR4

**Figure 1:**
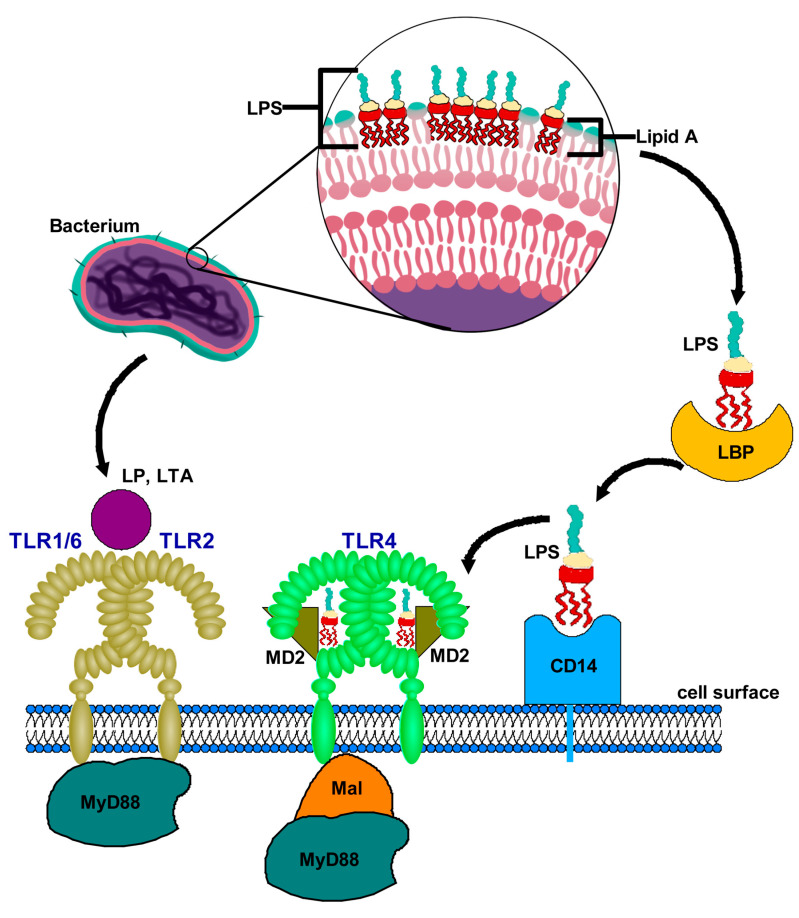
Ligand recognition by TLR4 and TLR2 receptors. Recognition of LPS by TLR4: LPS, released from the outer membrane of Gram-negative bacteria. LBP binds to LPS and presents it to CD14, which eventually leads to formation of TLR4-MD-2-LPS complex, resulting in dimerization of TLR4 subunits triggering TLR4 pathway. MD2 is necessary for TLR4 to bind to LPS and homodimerize. Recognition of LP, LTA by TLR2: TLR2 forms a heterodimer on the cell surface with co-receptors TLR1 or TLR6. The TLR2/TLR1 and TLR2/TLR6 heterodimers specifically bind LP, LTA released from Gram-positive bacteria. The ligand binding to heterodimer brings the intracellular TIR domains close to each other and initiate signaling. Abbreviations: LPS, lipopolysaccharide; LBP, LPS binding protein; LTA, lipoteichoic acid; LP, lipopeptide; MD2, myeloid differentiation factor 2; Mal, MyD88-adapter-like; MyD88, myeloid differentiation 88 protein.

TLR2 recognizes a wide range of ligands, which can be broadly categorized into microbial and endogenous components (Figure 1). Microbial ligands include triacylated and diacylated lipopeptides, peptidoglycans and lipoteichoic acid from bacteria, as well as components from fungi and viruses [21]. Endogenous ligands, such as heat shock proteins (HSPs) and high mobility group box 1 (HMGB1), can also activate TLR2, contributing to sterile inflammation and tissue repair processes [22]. The interaction of TLR2 with these ligands triggers intracellular signaling cascades, primarily through the MyD88-dependent pathway, leading to the activation of NF-κB and the production of proinflammatory cytokines [23].

TLR4 is primarily activated by LPS, a major component of the outer membrane of Gram-negative bacteria (Figure 1). The binding of LPS to TLR4 requires the presence of both the accessory protein CD14 and the MD-2 protein, which facilitates the formation of a signaling complex [24]. This interaction initiates a robust immune response characterized by the production of proinflammatory cytokines, chemokines, and the recruitment of immune cells to the site of infection. In addition to LPS, TLR4 can recognize a variety of other ligands, including endogenous molecules such as HSPs, fibronectin, and hyaluronic acid, which can be released during tissue injury or inflammation [25]. These interactions have also been implicated in chronic inflammatory responses in various diseases, including cancer and autoimmune disorders [26].

### 3.3. Signaling Pathways of TLR2 and TLR4

Upon ligand binding, TLR2 primarily activates the MyD88-dependent signaling pathway, which leads to the recruitment of various signaling molecules, including interleukin-1 receptor-associated kinase (IRAK) and tumor necrosis factor receptor-associated factor 6 (TRAF6) [23,27]. This cascade results in the activation of NF-κB and mitogen-activated protein kinases (MAPKs), which are crucial for the transcription of proinflammatory cytokines such as TNF-α, IL-1β, and IL-6 [28]. Additionally, TLR2 can also activate the TRIF-dependent pathway, although this occurs less frequently. This pathway is associated with the production of type I interferons and is particularly important in the context of viral infections [29]. The ability of TLR2 to engage multiple signaling pathways underscores its versatility in modulating immune responses.

TLR4 signaling also occurs through two pathways: the MyD88-dependent pathway and the TRIF-dependent pathway. The MyD88-dependent pathway is activated upon LPS binding and leads to the recruitment of IRAK and TRAF6, resulting in NF-κB activation and proinflammatory cytokine production [30]. This pathway is critical for the early immune response to bacterial infections. The TRIF-dependent pathway is also activated by LPS and involves the recruitment of TRIF, leading to the activation of interferon regulatory factors (IRFs) and the subsequent production of type I interferons [31]. This pathway is particularly important for antiviral responses and the regulation of adaptive immunity. The dual signaling capabilities of TLR4 allow for a robust and multifaceted immune response to a variety of pathogens.

Recent studies have highlighted the crosstalk between the TLR2 and TLR4 signaling pathways, suggesting that simultaneous activation of both receptors can enhance the immune response. For example, the costimulation of TLR2 and TLR4 in macrophages synergistically increases the production of proinflammatory cytokines, amplifying the overall immune response to infections [32]. This crosstalk may also contribute to the development of chronic inflammatory conditions, where persistent activation of TLRs can lead to tissue damage and disease progression [33].

### 3.4. Role of TLR2 and TLR4 in Fungal Recognition and RVVC

**Figure 2:**
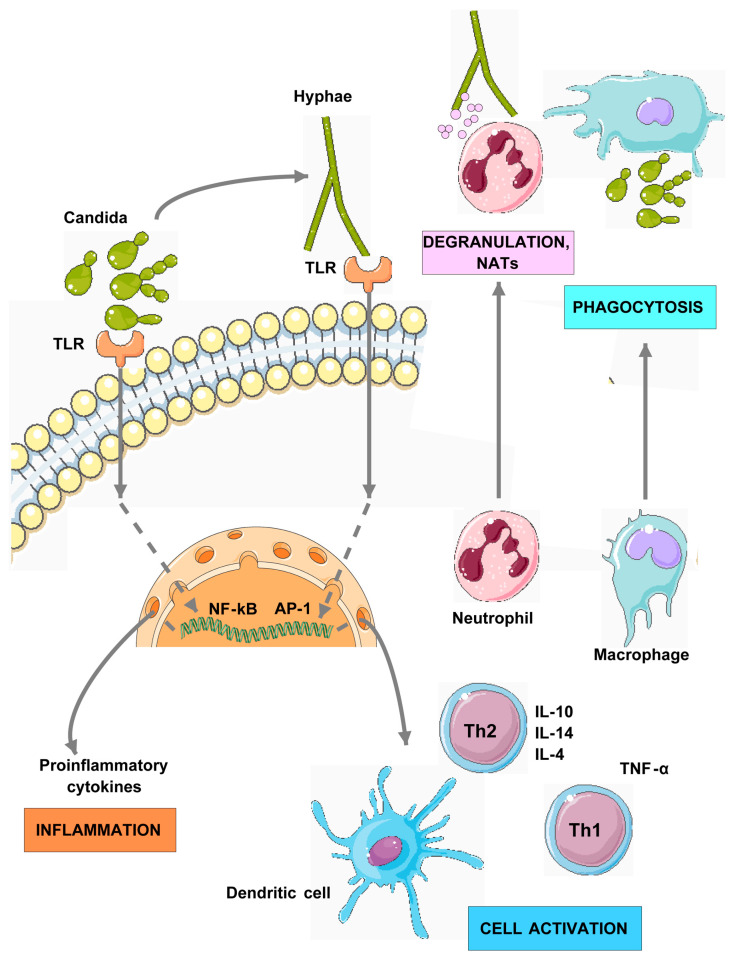
*Candida* goes through a reversible yeast-to-hyphae transformation during infection, which alters the kind of surface carbohydrates and activates TLRs. In response, inflammatory immune mediators such as cytokines, antimicrobial peptides, chemokines, and damage-associated molecular patterns are produced, which then recruit innate immune cells such as macrophages, dendritic cells, and neutrophils. These cells also sense PAMPs via PRRs on their surfaces, bind to the pathogen, and induce phagocytosis of yeast cells. Since *Candida* hyphae are too large to be phagocytosed, neutrophils use extracellular killing mechanisms by releasing toxic granule contents and releasing Neutrophil Extracellular Traps (NETs), a mesh of DNA, histones, and antimicrobial proteins, that kill hyphae extracellularly. *C. albicans* interaction with TLRs also activates T-helper cells. More specifically, *C. albicans* interaction with TLR4 was found to promote Th1 responses, whereas interaction with TLR2 enhances Th2 responses [44,45]. As a result, interactions between distinct fungal structures and different TLRs elicit different responses, polarizing T-helper response toward Th1 or Th2 based on the cytokine profile that could be generated upon these interactions. Cells depicted were provided by Servier Medical Art (https://smart.servier.com), licensed under CC BY 4.0 (https://creativecommons.org/licenses/by/4.0/).

TLRs are integral to the ability of the innate immune system to detect fungal infections. Specifically, TLR2 and TLR4 have been shown to recognize components of the *Candida* cell wall, such as β-glucans and mannan, leading to the activation of inflammatory pathways and cytokine production [6,34] Figure 2. This recognition is vital for the recruitment of immune cells to the site of infection, facilitating phagocytosis and the subsequent clearance of the pathogen [35].

TLR2 recognizes phospholipomannans [36], which leads to the local production of proinflammatory cytokines such as IL-1β and IL-6, which are key mediators of immune cell recruitment to the site of infection [11,37]. Marıa Soledad Mirο and coworkers used TLR2-deficient mice to study the early innate immune response during VVC. It was discovered that the absence of TLR2-mediated signaling predisposed subjects to an elevated initial vaginal fungal burden, which was followed by effective clearance but not complete pathogen eradication [38,38]. Similarly, TLR4 responds to O-linked mannans [39], contributing to the inflammatory response. Genetic polymorphisms in these receptors can influence an individual’s susceptibility to RVVC [12].

Research indicates that TLR signaling not only promotes protective immune responses but also contributes to inflammatory pathology, potentially exacerbating the symptoms of RVVC [34,40]. This dual role underscores the complexity of TLR-mediated responses in fungal infections, where an excessive immune response may result in tissue damage and persistent symptoms.

The activation of TLRs leads to the production of various cytokines that orchestrate the immune response against *Candida* infections. In patients with RVVC, an exaggerated cytokine response, particularly in the presence of hyphae, has been observed, which is formed almost exclusively by *C. albicans* [6,20]. This heightened inflammatory response is believed to contribute to the symptoms associated with RVVC, negatively impacting quality of life.

Cytokines such as IL-1β and TNF-α are crucial for recruiting neutrophils and macrophages to the site of infection, promoting phagocytosis and fungal clearance [41]. However, excessive cytokine production can lead to chronic inflammation, perpetuating the reinfection-inflammation cycle, which is characteristic of RVVC [42,43]. Understanding the balance between protective and pathological immune responses is essential for the development of targeted therapies for RVVC.

## 4. Discussion

### 4.1. Genetic Polymorphisms and Immune Response

Single-nucleotide polymorphisms (SNPs) strongly influence innate immune responses to pathogenic challenges and disease outcomes; therefore, individual susceptibility to infections varies, with some people being predisposed to certain infections and others being more resistant [46]. SNPs in genes involved in immune pathways for cytokine production, cell signalling, and cell adhesion pathways have been previously associated with the susceptibility to RVVC [47]. Also, host genetic variants in PRRs have long been thought to impair the antifungal immune response in RVVC patients. Genetic variations, including polymorphisms in TLRs and mannose-binding lectin, which may increase susceptibility to VVC [20], have been observed in women with RVVC [4,48]. Single-nucleotide polymorphisms and other genetic variations that impact critical signaling proteins in the host may accelerate the development of VVC and raise susceptibility to RVVC [20,49]. Genetic variants in pattern recognition receptors or signal transducers have been discovered to hinder the antifungal immune response in RVVC patients [20]. Variants in TLR2 and TLR4 have been associated with altered immune responses to *Candida* infections, suggesting that genetic predisposition plays a role in the pathogenesis of RVVC [50,51]. For example, certain TLR polymorphisms may impair the recognition of *Candida* components, leading to inadequate immune responses and increased susceptibility to recurrent infections [12].

### 4.2. Polymorphisms in TLR4

The TLR4 gene is known to harbor several SNPs, with the most studied being Asp299Gly (rs4986790) and Thr399Ile (rs4986791). These two polymorphisms are in linkage disequilibrium. Linkage disequilibrium refers to the nonrandom association of alleles at different loci, which can often be observed in genetic studies. Several studies have reported that the Asp299Gly and Thr399Ile polymorphisms frequently cosegregate. Senhaji et al. conducted a meta-analysis that confirmed the presence of linkage disequilibrium between these two TLR4 polymorphisms, noting that they are often coinherited together as a haplotype [52]. This finding is supported by the work of Ferwerda et al., who discussed how the proinflammatory phenotype associated with the Asp299Gly allele may have evolutionary implications, further suggesting the coinheritance of these alleles [53]. The TLR4 Asp299Gly Thr399Ile haplotype has been reported to alter the leucine-rich repeat region of the receptor and decrease the efficiency of ligand recognition [54].

Numerous studies have revealed a connection between TLR4 polymorphisms and susceptibility to infections. For example, the Asp299Gly polymorphism has been linked to a hypo-responsive state to LPS [54], resulting in increased susceptibility to infections caused by Gram-negative bacteria [55,56]. This hypo responsiveness is particularly evident in individuals with the Asp299Gly variant, who exhibit diminished production of proinflammatory cytokines upon LPS stimulation [55,57]. Individuals carrying the Asp299Gly variant are at increased risk of developing severe infections, such as those caused by *Haemophilus influenzae* and *Mycobacterium tuberculosis* [56]. A recent meta-analysis revealed that the TLR4 polymorphic locus rs10759932, which is located in an upstream regulatory region of the TLR4 gene, increased the risk of pulmonary tuberculosis [58]. The G allele of rs4986790 (Asp299Gly mutation) is also an independent risk factor for pulmonary tuberculosis [58].

Two other studies have demonstrated a link between this SNP and an increased risk of septic shock due to infection by Gram-negative bacteria [59,60]. The TLR4 Asp299Gly haplotype has also been associated with an increased incidence of systemic inflammatory response syndrome [61]. Ziakas et al. conducted a meta-analysis that highlighted the association of the TLR4 896 A>G and 1196 C>T SNPs with an increased risk for various infections, including malaria and other parasitic diseases [62]. Both the TLR4-Asp299Gly and the TLR4-Thr399Ile variants confer an increased risk of severe malaria in Ghanaian children, linking these SNPs to disease manifestation [63]. Rasouli et al. further elucidated the role of TLR4 polymorphisms in visceral leishmaniasis, demonstrating a higher prevalence of certain SNPs among affected individuals [64].

The strongest association between TLR4 polymorphisms and disease susceptibility has been demonstrated with respiratory syncytial virus (RSV) infection, with newborns heterozygous for Asp299Gly and Thr399Ile being more susceptible to infection [65]. Silva et al. reinforced this notion through a comprehensive meta-analysis, indicating that the TLR4 896A/G polymorphism is linked to a diverse spectrum of infections, emphasizing its complex role in immune response modulation [66].

The mechanisms by which TLR4 SNPs influence disease susceptibility are multifaceted. The Asp299Gly polymorphism has been shown to alter TLR4 signaling pathways, leading to decreased NF-κB activation and altered cytokine production [67]. This alteration can result in a diminished inflammatory response, which may predispose individuals to infections. Hold et al. demonstrated that both the Asp299Gly and Thr399Ile SNPs can upregulate the expression of TRIF-dependent genes, which play a catalytic role in the immune response to pathogens [57]. These findings suggest that while these polymorphisms may confer susceptibility to certain infections, they may also enhance responses to others, thus demonstrating the intricate balance of immune regulation. The presence of the Asp299Gly variant has been associated with reduced responsiveness to lipopolysaccharide (LPS), suggesting a hyporesponsive state in carriers, which is corroborated by evidence of diminished NF-κB activation upon TLR4 stimulation [68,69]. In vitro studies have shown that TLR4 variants may form less efficient signaling complexes or misfold, preventing optimal downstream signaling [69,70]. The Thr399Ile polymorphism has been shown to affect the receptor’s ligand binding capability and subsequent signal transduction efficiency [71,72].

### 4.3. TLR4 Polymorphisms and Fungal Infections

TLR4 variants also influence antifungal immune responses. The TLR4 Asp299Gly Thr399Ile haplotype is associated with the development of invasive pulmonary aspergillosis (IPA) in donors of allogeneic stem cell transplantation (HSCT) [73]. However, the exact mechanism remains unknown, particularly since no fungal ligands have been identified to date. One proposed mechanism is that variations in the TLR4 gene alter cytokine production, which may affect the inflammatory response and the clearance of fungal infections [12].

**Figure 3:**
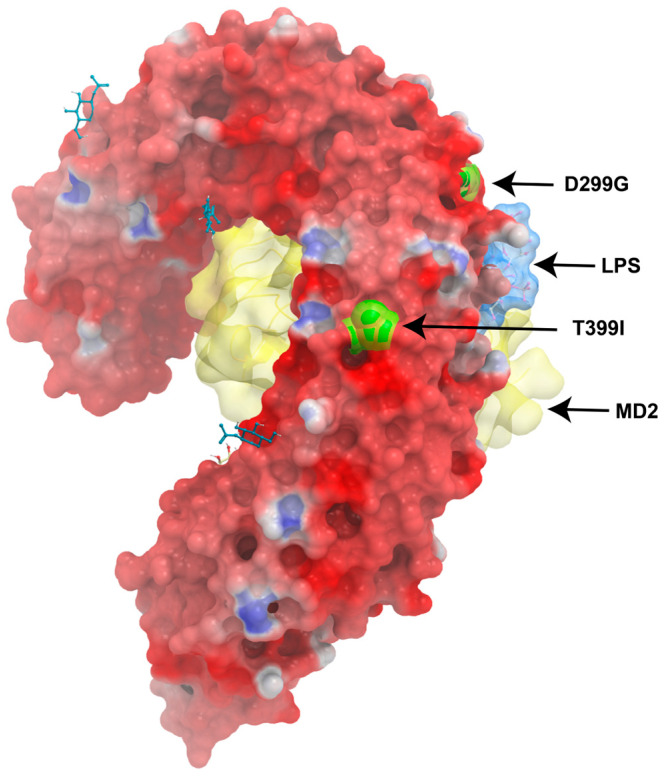
Three-dimensional structure of TLR4 and localization of its major polymorphisms.

The crystal structure of TLR4 Asp299Gly/Thr399Ile has been solved as a complex with MD-2 and LPS [74,75]. Compared with the wild-type TLR4/MD-2/LPS complex structure, the overall arrangements of the two complexes were similar, and topical differences were present around only the Asp299Gly SNP site, which induced a structural change that modulated the surface properties of TLR4 (Figure 3). This effect may be more apparent upon stimulation of TLR4 with ligands with weak agonistic activity. The impact of the Thr399Ile change was minor, as nearly no structural differences were observed [75].

### 4.4. Polymorphisms in TLR2

TLR2, as a heterodimer with TLR1 or TLR6, recognizes common bacterial motifs, including peptidoglycans, lipopeptides, glycosylphosphatidylinositol-linked proteins and zymosan. Dysregulation of TLR2 signaling due to genetic polymorphisms can lead to altered immune responses, potentially increasing susceptibility to infectious diseases [76].

Several single-nucleotide polymorphisms (SNPs) have been identified in the TLR2 gene, with the most studied being Arg677Trp (rs121917864) and Arg753Gln (rs5743708). These polymorphisms can affect the receptor’s ability to recognize PAMPs and modulate immune responses [76]. For example, the Arg677Trp variant has been associated with altered cytokine production in response to bacterial infections, suggesting a potential link to increased susceptibility to infectious diseases [77]. Arg677Trp is common in African and Asian populations but is almost absent among Caucasian populations [46]. In vitro, this SNP has been shown to inhibit both *Mycobacterium leprae-* and *Mycobacterium tuberculosis*-mediated NF-κB activation and production [78]. In a studied Korean population and a Tunisian population, this SNP was associated with leprosy [79] and susceptibility to tuberculosis [80], respectively. Moreover, a study conducted in Iran revealed that the TLR2 Arg677Trp polymorphism was associated with a greater likelihood of infection among individuals exposed to *Mycobacterium tuberculosis* [81]. A meta-analysis revealed that the TLR2 Arg677Trp polymorphism was associated with an increased risk of severe periodontitis, confirming its role in modulating the immune response to oral pathogens [82].

Studies have also revealed that the TLR2 Arg753Gln polymorphism is associated with increased susceptibility to tuberculosis and other infections. In this context, Bhanothu et al. reported that the TLR2 Arg753Gln variant is associated with the susceptibility of females to tuberculosis, suggesting that this SNP may influence immune responses to mycobacterial infections [83,84]. This polymorphism was also associated with an increased risk of developing tuberculosis in a Turkish population [85], along with a significantly increased risk for some individuals to develop infective endocarditis [86]. Additionally, TLR2 polymorphisms have been studied in the context of viral infections. Research has shown that certain TLR2 variants may influence the immune response to viruses such as dengue and HIV, potentially affecting disease outcomes [87]. Kang et al. demonstrated that homozygosity for the TLR2 Arg753Gln SNP is a risk factor for cytomegalovirus disease following liver transplantation, indicating its potential role in modulating immune responses in transplant patients [88]. Another study indicated that TLR2 polymorphisms are associated with the severity of dengue virus infection, suggesting that genetic variations in TLR2 may impact the host’s ability to control viral replication [89].

**Figure 4:**
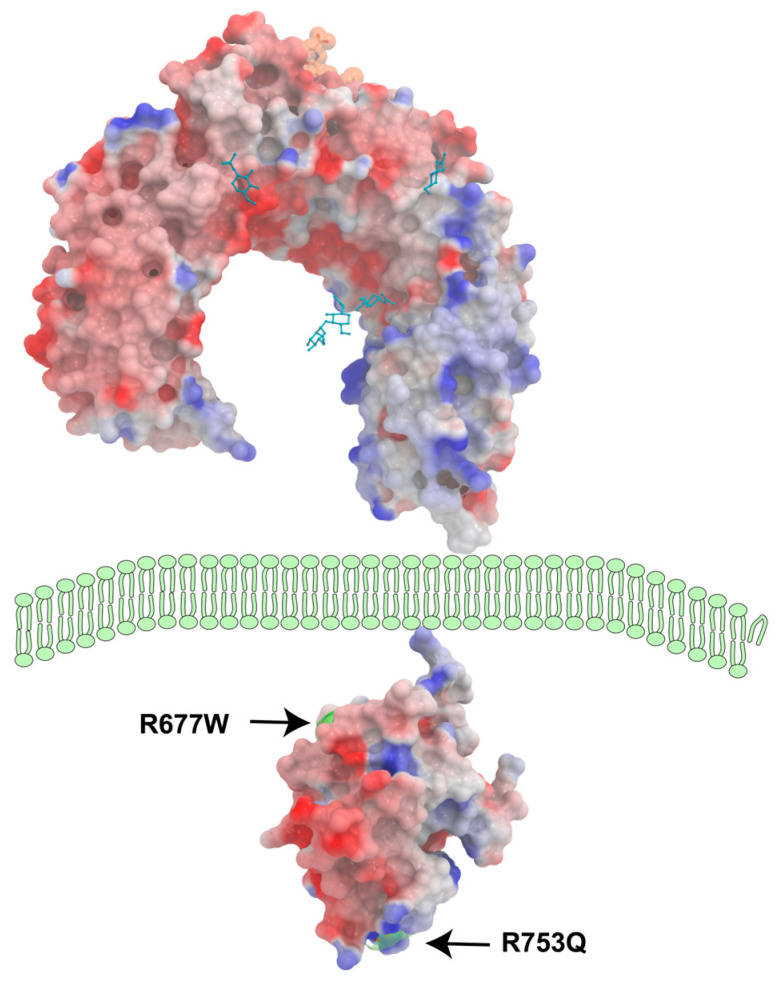
Three-dimensional structure of TLR2 and localization of its major polymorphisms.

The Arg677Trp and Arg753Gln polymorphisms in TLR2 have been implicated in modifying the receptor’s function (Figure 4), thereby affecting the extent of downstream signaling activation. Specifically:

1. Arg677Trp (rs121917864): This polymorphism is believed to potentially alter the affinity of TLR2 for its ligands. Research indicates that this variant may influence the strength of TLR2-mediated responses, which can modulate the activation of NF-κB. It has been demonstrated that individuals carrying this variant exhibit different susceptibility profiles to infectious diseases such as tuberculosis and chronic inflammatory conditions, suggesting that the intrinsic signaling efficiency may be altered [81,90].
2. Arg753Gln (rs5743708): This polymorphism similarly affects TLR2 function. Studies suggest that Arg753Gln may lead to decreased NF-κB activation in response to TLR2 ligands, thereby reducing the production of key inflammatory cytokines [91]. This variant may also impact the cross-talk between TLR2 and other signaling pathways involved in IRF activation, which can indirectly regulate the innate immune response [92,93].

The ability of TLR2 polymorphisms to influence the intensity of the inflammatory response is particularly important in diseases characterized by chronic inflammation, such as chronic obstructive pulmonary disease (COPD), rheumatoid arthritis, and diabetes [91,94,95]. The differential regulation of NF-κB and IRF pathways by TLR2 variants shapes the overall immune response and can determine susceptibility to inflammation-related diseases.

### 4.5. TLR2 Polymorphisms and Fungal Infections

The role of TLR2 in fungal infections has also been explored, particularly in the context of invasive fungal diseases. Studies have brought to light that TLR2 is involved in the recognition of fungal components, such as β-glucans, and plays a critical role in the immune response to fungi such as *C. albicans* and Aspergillus species [96]. Polymorphisms in the TLR2 gene may affect susceptibility to these infections, with certain variants associated with an increased risk of invasive fungal disease in immunocompromised patients [97]. For example, the R753Q TLR2 polymorphism increased the risk for candidaemia in a limited study through decreased IFN-γ and IL-8 levels [98]. Similarly, a study examining TLR2 polymorphisms in hematopoietic stem cell transplant recipients revealed that specific genetic variants were linked to an increased risk of developing invasive aspergillosis, underscoring the importance of TLR2 in antifungal immunity [99].

The Pro631His (rs5743704) SNP in TLR2 has been implicated in the development of idiopathic recurrent vulvovaginal candidiasis (RVVC). More specifically, the TLR2 Pro631His polymorphism was associated with an almost 3-fold increase in susceptibility to RVVC [100]. Moreover, TLR2 deficiency influences susceptibility to systemic candidiasis in mice [101].

### 4.6. Polymorphisms in TLRs, Other than TLR2 and TLR4, and Fungal Infections

Genetic variations in the TLR1, TLR3, TLR5 and TLR9 TLRs have also been proposed as risk factors for fungal disease. TLR1 appears to be a key reservoir of genetic variation, which increases candidaemia susceptibility [102]. TLR9 mutation rs5743836 (in the promoter region) is linked to the development of allergic bronchopulmonary aspergillosis (ABPA) [103]. Another link includes TLR5, where an SNP resulting in an early stop codon (Arg392X) has been demonstrated to impair flagellin recognition [104]. The presence of this variation in HSCT recipients was related to the development of IPA [104], indicating that TLR5 has a crucial antifungal function. Despite being known as a prototype receptor for double-stranded RNA, TLR3 has been linked to fungus identification and the activation of adaptive immune responses. The regulatory variation rs3775296 in TLR3 was found to increase the risk of IPA following HSCT [105]. The non-synonymous SNP rs3775291 (Leu412Phe) in TLR3 has been identified more frequently in patients suffering from chronic mucocutaneous candidiasis (CMC) [106].

### 4.7. Interactions with Other Immune Components

The immune response to *Candida* spp. is not solely dependent on TLRs; other components of the innate immune system, such as C-type lectin receptors (CLRs), also play a significant role. CLRs, including Dectin-1, work in concert with TLRs to enhance the recognition and clearance of *Candida* [42,107]. The collaboration between TLRs and CLRs is vital for a comprehensive immune response, as they recognize different fungal components and activate distinct signaling pathways. In this context, genetic factors, such as those related to the mannose-binding lectin (MBL) pathway, increase the risk of RVVC. MBL deficiency has been shown to increase susceptibility to RVVC, highlighting the importance of a well-coordinated immune response in preventing recurrent infections [100]. The interplay between these genetic factors and the immune system underscores the need for a comprehensive understanding of the immunological landscape in RVVC patients.

Moreover, the interaction between TLRs and other PRRs, such as Dectin-1, is crucial for a robust antifungal response. Dectin-1 recognizes β-glucans, while TLRs detect other fungal components, creating a synergistic effect that enhances the immune response against *Candida* [42,108]. This interplay underscores the importance of a well-functioning innate immune system in preventing RVVC.

Furthermore, the role of the inflammasome in the immune response to *Candida* spp. has gained attention. The NLRP3 inflammasome, for example, is activated in response to *Candida* spp. infection, leading to the processing of proinflammatory cytokines [41]. This interaction highlights the complexity of the immune response, where multiple pathways converge to combat fungal infections.

## 5. Clinical Implications and Future Research Directions

The identification of specific TLR polymorphisms associated with RVVC risk can aid in the development of targeted treatments and prevention strategies. Understanding how genetic variability in immune receptors affects infection susceptibility opens possibilities for personalized therapeutic approaches, potentially using immunomodulators or antifungal therapies tailored to individual genetic profiles. TLRs themselves are also potential targets for therapeutic interventions. TLRs can be both friends and foes since improperly regulated TLR signaling can result either in the overactivation of immune responses, leading to pathological inflammation, or in diminished inflammatory responses, which may predispose individuals to infections. Recent efforts have focused on the development of both TLR antagonists as anti-inflammatory drug candidates and TLR agonists as immunotherapeutics [84,109]. In this context, SMP-105 TLR2 agonist has been proposed as an agent for the treatment of bladder cancer [110] and TAK-242 TLR4 antagonist has been shown to exhibit antitumor effect [111]. Further research is warranted to investigate other polymorphisms in TLR-related pathways and their potential interactions with environmental factors that may contribute to RVVC, as well as to elucidate how these pathways might be therapeutically targeted to prevent recurrence.

## 6. Conclusions

Since polymorphisms in the TLR signaling pathway can affect susceptibility to infections, it is likely that in the near future these variations will be used as a predictive and preventive tool in medicine. Current literature suggests that TLR signaling not only promotes protective immune responses but also contributes to inflammatory pathology, potentially exacerbating the symptoms of RVVC. In this regard, certain TLR polymorphisms may impair the recognition of *Candida* components, leading to inadequate immune responses and increased susceptibility to recurrent infections. On the other hand, the activation of TLRs leads to the production of various cytokines that orchestrate the immune response against fungal infections. In patients with RVVC, an exaggerated cytokine response, particularly in the presence of hyphae, has been observed. This excessive cytokine production can lead to chronic inflammation, perpetuating the reinfection-inflammation cycle, which is characteristic of RVVC, negatively impacting the quality of life of the patients. This dual role underscores the complexity of TLR-mediated responses in fungal infections, where an excessive immune response may result in tissue damage and persistent symptoms.

This literature review summarizes current insights into the genetic underpinnings of RVVC, focusing on polymorphisms in TLR2 and TLR4 as significant factors in the host immune response, and highlights future directions for research and clinical practice to improve outcomes for those affected by RVVC.

Understanding the role of TLR polymorphism and its association with RVVC may lead to novel immune modulation techniques employing medications or vaccines that target TLR activation pathways and polymorphic TLRs. Such advancements may lead to personalized medicine strategies that take into account each patient’s unique genetic makeup.

## Data Availability

No new data were created or analyzed in this study. Data sharing is not applicable to this article.

## References

[1] Kalia N., Singh J., Kaur M. (2019). Immunopathology of Recurrent Vulvovaginal Infections: New Aspects and Research Directions. Front. Immunol..

[2] Binsaad A.J.A., Al-Abd N. (2021). The Prevalence of Vulvovaginal Candidiasis (Vvc) Among Women Suffering Vaginitis Attended a Private Gynecological Clinic, Aden-Yemen. Electron. J. Univ. Aden Basic. Appl. Sci..

[3] Salama O.E., Gerstein A.C. (2022). Differential Response of *Candida* Species Morphologies and Isolates to Fluconazole and Boric Acid. Antimicrob. Agents Chemother..

[4] Denning D.W., Kneale M., Sobel J.D., Rautemaa-Richardson R. (2018). Global burden of recurrent vulvovaginal candidiasis: A systematic review. Lancet Infect. Dis..

[5] Sobel J.D. (2016). Recurrent vulvovaginal candidiasis. Am. J. Obs. Gynecol..

[6] Rosati D., Bruno M., Jaeger M., Kullberg B.J., van de Veerdonk F., Netea M.G., Oever J.T. (2020). An Exaggerated Monocyte-Derived Cytokine Response to *Candida* Hyphae in Patients with Recurrent Vulvovaginal Candidiasis. J. Infect. Dis..

[7] Zhu Y., Li T., Fan S.R., Liu P., Liang Y., Liu P. (2016). Health-Related Quality of Life as Measured with the Short-Form 36 (SF-36) Questionnaire in Patients with Recurrent Vulvovaginal Candidiasis. Health Qual. Life Outcomes.

[8] Blostein F., Levin-Sparenberg E., Wagner J., Foxman B. (2017). Recurrent vulvovaginal candidiasis. Ann. Epidemiol..

[9] Foxman B., Muraglia R., Dietz J.P., Sobel J.D., Wagner J. (2013). Prevalence of recurrent vulvovaginal candidiasis in 5 European countries and the United States: Results from an internet panel survey. J. Low. Genit. Tract Dis..

[10] Willems H.M.E., Ahmed S.S., Liu J., Xu Z., Peters B.M. (2020). Vulvovaginal Candidiasis: A Current Understanding and Burning Questions. J. Fungi.

[11] Ardizzoni A., Wheeler R.T., Pericolini E. (2021). It Takes Two to Tango: How a Dysregulation of the Innate Immunity, Coupled with Candida Virulence, Triggers VVC Onset. Front. Microbiol..

[12] Smeekens S.P., Frank L.v.d.V., Kullberg B.J., Netea M.G. (2013). Genetic Susceptibility To *Candida* Infections. Embo Mol. Med..

[13] Chen Y.-H., Wu K.H., Wu H.P. (2024). Unraveling the Complexities of Toll-like Receptors: From Molecular Mechanisms to Clinical Applications. Int. J. Mol. Sci..

[14] Duan T., Du Y., Xing C., Wang H.Y., Wang R.F. (2022). Toll-like Receptor Signaling and Its Role in Cell-Mediated Immunity. Front. Immunol..

[15] Kawai T., Akira S. (2010). The Role of Pattern-Recognition Receptors in Innate Immunity: Update on Toll-like Receptors. Nat. Immunol..

[16] Kim S.-N., Joe Y., Surh Y.J., Chung H.T. (2018). Differential Regulation of Toll-like Receptor-Mediated Cytokine Production by Unfolded Protein Response. Oxidative Med. Cell. Longev..

[17] Kubarenko A.V., Ranjan S., Çolak E., George J., Frank M., Weber A.N. (2010). Comprehensive Modeling and Functional Analysis of Toll-like Receptor Ligand-recognition Domains. Protein Sci..

[18] Takeda K., Akira S. (2015). Toll-like Receptors. Curr. Protoc. Immunol..

[19] Goncalves B., Ferreira C., Alves C.T., Henriques M., Azeredo J., Silva S. (2016). Vulvovaginal candidiasis: Epidemiology, microbiology and risk factors. Crit. Rev. Microbiol..

[20] Rosati D., Bruno M., Jaeger M., ten Oever J., Netea M.G. (2020). Recurrent Vulvovaginal Candidiasis: An Immunological Perspective. Microorganisms.

[21] Rasaei R., Sarodaya N., Kim K.S., Ramakrishna S., Hong S.H. (2020). Importance of Deubiquitination in Macrophage-Mediated Viral Response and Inflammation. Int. J. Mol. Sci..

[22] Chen L., Yu J. (2016). Modulation of Toll-like Receptor Signaling in Innate Immunity by Natural Products. Int. Immunopharmacol..

[23] O’Neill L.A., Bowie A.G. (2007). The family of five: TIR-domain-containing adaptors in Toll-like receptor signalling. Nat. Rev. Immunol..

[24] Guo J., Liao M., Wang J. (2021). TLR4 Signaling in the Development of Colitis-Associated Cancer and Its Possible Interplay with microRNA-155. Cell Commun. Signal..

[25] Shi J.h., Sun S.C. (2018). Tumor Necrosis Factor Receptor-Associated Factor Regulation of Nuclear Factor ΚB and Mitogen-Activated Protein Kinase Pathways. Front. Immunol..

[26] Gozalbo D. (2016). Role of Toll-like Receptors in Systemic *Candida albicans* Infections. Front. Biosci..

[27] Marongiu L., Gornati L., Artuso I., Zanoni I., Granucci F. (2019). Below the Surface: The Inner Lives of TLR4 and TLR9. J. Leukoc. Biol..

[28] Behzadi P., García-Perdomo H.A., Karpiński T.M. (2021). Toll-like Receptors: General Molecular and Structural Biology. J. Immunol. Res..

[29] Abdul-Cader M.S., Amarasinghe A., Abdul-Careem M.F. (2016). Activation of Toll-like Receptor Signaling Pathways Leading to Nitric oxide-Mediated Antiviral Responses. Arch. Virol..

[30] McKeown-Longo P.J., Higgins P.J. (2017). Integration of Canonical and Noncanonical Pathways in TLR4 Signaling: Complex Regulation of the Wound Repair Program. Adv. Wound Care.

[31] Xu R.h., Li Y., Liu Y., Qu J., Cao W., Zhang E., He J., Cai Z. (2020). How Are McPip1 and Cytokines Mutually Regulated in Cancer-Related Immunity?. Protein Cell.

[32] Scheenstra M.R., Harten R.M.v., Veldhuizen E.J., Haagsman H.P., Coorens M. (2020). Cathelicidins Modulate TLR-Activation and Inflammation. Front. Immunol..

[33] Reed S.G., Hsu F.-C., Carter D., Orr M.T. (2016). The Science of Vaccine Adjuvants: Advances in TLR4 Ligand Adjuvants. Curr. Opin. Immunol..

[34] Cunha C., Romani L., Carvalho A. (2010). Cracking the Toll-like Receptor Code in Fungal Infections. Expert. Rev. Anti-Infect. Ther..

[35] Nedovic B., Posteraro B., Leoncini E., Ruggeri A., Amore R., Ricciardi W., Boccia S. (2014). Mannose-Binding Lectin Codon 54 Gene Polymorphism and Vulvovaginal Candidiasis: A Systematic Review and Meta-Analysis. BioMed Res. Int..

[36] Jouault T., Ibata-Ombetta S., Takeuchi O., Trinel P.A., Sacchetti P., Lefebvre P., Akira S., Poulain D. (2003). *Candida albicans* phospholipomannan is sensed through toll-like receptors. J. Infect. Dis..

[37] Yano J., Peters B.M., Noverr M.C., Fidel P.L. (2018). Novel Mechanism Behind the Immunopathogenesis of Vulvovaginal Candidiasis: “Neutrophil Anergy”. Infect. Immun..

[38] Miro M.S., Rodriguez E., Vigezzi C., Icely P.A., Garcia L.N., Peinetti N., Maldonado C.A., Riera F.O., Caeiro J.P., Sotomayor C.E. (2017). Contribution of TLR2 pathway in the pathogenesis of vulvovaginal candidiasis. Pathog. Dis..

[39] Netea M.G., Gow N.A., Munro C.A., Bates S., Collins C., Ferwerda G., Hobson R.P., Bertram G., Hughes H.B., Jansen T. (2006). Immune sensing of *Candida albicans* requires cooperative recognition of mannans and glucans by lectin and Toll-like receptors. J. Clin. Investig..

[40] van de Veerdonk F.L., Joosten L.A., Netea M.G. (2015). The interplay between inflammasome activation and antifungal host defense. Immunol. Rev..

[41] Joly S., Sutterwala F.S. (2010). Fungal Pathogen Recognition by the NLRP3 Inflammasome. Virulence.

[42] Bao M.-Y., Li M., Bu Q.-R., Yang Y., Song H., Wang C., Wang T., Li N. (2023). The Effect of Herbal Medicine in Innate Immunity to *Candida albicans*. Front. Immunol..

[43] Cunha C., Carvalho A., Esposito A., Bistoni F., Romani L. (2012). DAMP Signaling in Fungal Infections and Diseases. Front. Immunol..

[44] Netea M.G., Van der Graaf C., Van der Meer J.W., Kullberg B.J. (2004). Recognition of fungal pathogens by Toll-like receptors. Eur. J. Clin. Microbiol. Infect. Dis..

[45] Romani L., Montagnoli C., Bozza S., Perruccio K., Spreca A., Allavena P., Verbeek S., Calderone R.A., Bistoni F., Puccetti P. (2004). The exploitation of distinct recognition receptors in dendritic cells determines the full range of host immune relationships with *Candida albicans*. Int. Immunol..

[46] Skevaki C., Pararas M., Kostelidou K., Tsakris A., Routsias J.G. (2015). Single nucleotide polymorphisms of Toll-like receptors and susceptibility to infectious diseases. Clin. Exp. Immunol..

[47] Omosa-Manyonyi G.S., Ponce I.R., Rosati D., Bruno M., Kamau N.W., Obimbo M.M., Jaeger M., van der Ven A., Netea M.G., Kumar V. (2025). Genetic susceptibility to recurrent vulvovaginal candidiasis in an African population from Nairobi, Kenya. Sci. Rep..

[48] Babula O., Lazdane G., Kroica J., Linhares I.M., Ledger W.J., Witkin S.S. (2005). Frequency of interleukin-4 (IL-4) -589 gene polymorphism and vaginal concentrations of IL-4, nitric oxide, and mannose-binding lectin in women with recurrent vulvovaginal candidiasis. Clin. Infect. Dis..

[49] Bojang E., Ghuman H., Kumwenda P., Hall R.A. (2021). Immune Sensing of *Candida albicans*. J. Fungi.

[50] Plantinga T.S., Johnson M.D., Scott W.K., Joosten L.A.B., Meer J.W.M.v.d., Perfect J.R., Kullberg B.J., Netea M.G. (2012). Human Genetic Susceptibility To *Candida* infections. Med. Mycol..

[51] Zahedi N., Kenari S.A., Mohseni S., Aslani N., Ansari S., Badali H. (2016). Is Human Dectin-1 Y238X Gene Polymorphism Related to Susceptibility to Recurrent Vulvovaginal Candidiasis?. Curr. Med. Mycol..

[52] Senhaji N., Diakite B., Serbati N., Zaid Y., Badre W., Nadifi S. (2014). Toll-like Receptor 4 Asp299Gly and Thr399Ile Polymorphisms: New Data and a Meta-Analysis. BMC Gastroenterol..

[53] Ferwerda B., McCall M., Alonso S., Giamarellos-Bourboulis E.J., Mouktaroudi M., Izagirre N., Syafruddin D., Kibiki G., Cristea T., Hijmans A. (2007). *TLR4* polymorphisms, Infectious Diseases, and Evolutionary Pressure During Migration of Modern Humans. Proc. Natl. Acad. Sci. USA.

[54] Arbour N.C., Lorenz E., Schutte B.C., Zabner J., Kline J.N., Jones M., Frees K., Watt J.L., Schwartz D.A. (2000). TLR4 mutations are associated with endotoxin hyporesponsiveness in humans. Nat. Genet..

[55] Long H., O’Connor B.P., Zemans R.L., Zhou X., Yang I.V., Schwartz D.A. (2014). The Toll-like receptor 4 polymorphism Asp299Gly but not Thr399Ile influences TLR4 signaling and function. PLoS ONE.

[56] Tenhu E., Terasjarvi J., Cruzeiro M.L., Savonius O., Rugemalira E., Roine I., He Q., Pelkonen T. (2020). Gene Polymorphisms of TLR4 and TLR9 and Haemophilus influenzae Meningitis in Angolan Children. Genes..

[57] Hold G.L., Berry S., Saunders K.A., Drew J., Mayer C., Brookes H., Gay N.J., El-Omar E.M., Bryant C.E. (2014). The TLR4 D299G and T399I SNPs are constitutively active to up-regulate expression of Trif-dependent genes. PLoS ONE.

[58] Muheremu A., Jiang J., Yakufu M., Aili A., Li L., Luo Z. (2022). Relationship between tool-like receptor 4 gene polymorphism and the susceptibility to pulmonary tuberculosis. Am. J. Transl. Res..

[59] Agnese D.M., Calvano J.E., Hahm S.J., Coyle S.M., Corbett S.A., Calvano S.E., Lowry S.F. (2002). Human toll-like receptor 4 mutations but not CD14 polymorphisms are associated with an increased risk of gram-negative infections. J. Infect. Dis..

[60] Lorenz E., Mira J.P., Frees K.L., Schwartz D.A. (2002). Relevance of mutations in the TLR4 receptor in patients with gram-negative septic shock. Arch. Intern. Med..

[61] Child N.J., Yang I.A., Pulletz M.C., de Courcy-Golder K., Andrews A.L., Pappachan V.J., Holloway J.W. (2003). Polymorphisms in Toll-like receptor 4 and the systemic inflammatory response syndrome. Biochem. Soc. Trans..

[62] Ziakas P.D., Prodromou M.L., El Khoury J., Zintzaras E., Mylonakis E. (2013). The role of TLR4 896 A > G and 1196 C > T in susceptibility to infections: A review and meta-analysis of genetic association studies. PLoS ONE.

[63] Mockenhaupt F.P., Cramer J.P., Hamann L., Stegemann M.S., Eckert J., Oh N.R., Otchwemah R.N., Dietz E., Ehrhardt S., Schroder N.W. (2006). Toll-like receptor (TLR) polymorphisms in African children: Common TLR-4 variants predispose to severe malaria. Proc. Natl. Acad. Sci. USA.

[64] Rasouli M., Keshavarz M., Kalani M., Moravej A., Kiany S., Badiee P. (2012). Toll-like receptor 4 (TLR4) polymorphisms in Iranian patients with visceral leishmaniasis. Mol. Biol. Rep..

[65] Awomoyi A.A., Rallabhandi P., Pollin T.I., Lorenz E., Sztein M.B., Boukhvalova M.S., Hemming V.G., Blanco J.C., Vogel S.N. (2007). Association of TLR4 polymorphisms with symptomatic respiratory syncytial virus infection in high-risk infants and young children. J. Immunol..

[66] Silva M.J.A., Santana D.S., de Oliveira L.G., Monteiro E.O.L., Lima L. (2022). The relationship between 896A/G (rs4986790) polymorphism of TLR4 and infectious diseases: A meta-analysis. Front. Genet..

[67] Figueroa L., Xiong Y., Song C., Piao W., Vogel S.N., Medvedev A.E. (2012). The Asp299Gly polymorphism alters TLR4 signaling by interfering with recruitment of MyD88 and TRIF. J. Immunol..

[68] Bakaros E. (2023). Innate Immune Gene Polymorphisms and COVID-19 Prognosis. Viruses.

[69] Reilly F., Burke J.P., Lennon G., Kay E.W., McNamara D.A., Cullen G., Doherty G., Mulcahy H., Martin S.T., Winter D.C. (2021). A Case–control Study Examining the Association of *smad7* and *TLR* Single Nucleotide Polymorphisms on the Risk of Colorectal Cancer in Ulcerative Colitis. Color. Dis..

[70] Kesici G.G., Kaytez S.K., Özdaş T., Özdaş S. (2019). Association of Toll-like Receptor Polymorphisms With Nasal Polyposis. Ear Nose Throat J..

[71] Jiang S., Ma J., Ye S., Meaney C., Moore T.E., Pan S., Gao C. (2022). Associations Among Disseminated Intravascular Coagulation, Thrombocytopenia Cytokines/Chemokines and Genetic Polymorphisms of Toll-like Receptor 2/4 in Chinese Patients with Sepsis. J. Inflamm. Res..

[72] Tyurin Y.A., Шамсутдинoв А.Ф., Kalinin N.N., Шарифуллина А.А., Решетникoва И.Д. (2017). Association of Toll-like Cell Receptors TLR2 (p.Arg753GLN) and TLR4 (p.Asp299GLY) Polymorphisms with Indicators of General and Local Immunity in Patients With Atopic Dermatitis. J. Immunol. Res..

[73] Bochud P.Y., Chien J.W., Marr K.A., Leisenring W.M., Upton A., Janer M., Rodrigues S.D., Li S., Hansen J.A., Zhao L.P. (2008). Toll-like receptor 4 polymorphisms and aspergillosis in stem-cell transplantation. N. Engl. J. Med..

[74] Ohto U., Fukase K., Miyake K., Shimizu T. (2012). Structural basis of species-specific endotoxin sensing by innate immune receptor TLR4/MD-2. Proc. Natl. Acad. Sci. USA.

[75] Ohto U., Yamakawa N., Akashi-Takamura S., Miyake K., Shimizu T. (2012). Structural analyses of human Toll-like receptor 4 polymorphisms D299G and T399I. J. Biol. Chem..

[76] Wang M., Li L., Xiao S., Chen W., Hu F., Li F., Guo P., Chen X., Cai W., Tang X. (2021). The Association of TLR2, TLR3, and TLR9 Gene Polymorphisms With Susceptibility to Talaromycosis Among Han Chinese AIDS Patients in Guangdong. Front. Cell. Infect. Microbiol..

[77] Elloumi N., Tahri S., Fakhfakh R., Abida O., Mahfoudh N., Hachicha H., Marzouk S., Bahloul Z., Masmoudi H. (2022). Role of Innate Immune Receptors TLR4 and TLR2 Polymorphisms in Systemic Lupus Erythematosus Susceptibility. Ann. Hum. Genet..

[78] Kang T.J., Lee S.B., Chae G.T. (2002). A polymorphism in the toll-like receptor 2 is associated with IL-12 production from monocyte in lepromatous leprosy. Cytokine.

[79] Kang T.J., Chae G.T. (2001). Detection of Toll-like receptor 2 (TLR2) mutation in the lepromatous leprosy patients. FEMS Immunol. Med. Microbiol..

[80] Ben-Ali M., Barbouche M.R., Bousnina S., Chabbou A., Dellagi K. (2004). Toll-like receptor 2 Arg677Trp polymorphism is associated with susceptibility to tuberculosis in Tunisian patients. Clin. Diagn. Lab. Immunol..

[81] Davoodi H., Ghaemi E.A., Jamali A., Naeeme J.S., Shakeri F. (2018). Arg677Trp and Arg753Gln Polymorphisms in TLR2 Genes Detected in Patients with Tuberculosis in Golestan Province, Iran. Jundishapur J. Microbiol..

[82] Shan C., Abasijiang A., Wu Z.P., Wang T.T., Jin Z. (2020). Association of TLR-2 Gene Polymorphisms With the Risk of Periodontitis: A Meta-Analysis. Dis. Markers.

[83] Bhanothu V., Lakshmi V., Theophilus J., Rozati R., Badhini P., Vijayalaxmi B. (2015). Investigation of Toll-like Receptor-2 (2258g/A) and Interferon Gamma (+874t/A) Gene Polymorphisms Among Infertile Women with Female Genital Tuberculosis. PLoS ONE.

[84] Hu W., Spaink H.P. (2022). The Role of TLR2 in Infectious Diseases Caused by Mycobacteria: From Cell Biology to Therapeutic Target. Biology.

[85] Ogus A.C., Yoldas B., Ozdemir T., Uguz A., Olcen S., Keser I., Coskun M., Cilli A., Yegin O. (2004). The Arg753GLn polymorphism of the human toll-like receptor 2 gene in tuberculosis disease. Eur. Respir. J. Off. J. Eur. Soc. Clin. Respir. Physiol..

[86] Bustamante J., Tamayo E., Florez S., Telleria J.J., Bustamante E., Lopez J., San Roman J.A., Alvarez F.J. (2011). Toll-like receptor 2 R753Q polymorphisms are associated with an increased risk of infective endocarditis. Rev. Esp. Cardiol..

[87] Masin P.S., Visentin H.A., Elpidio L.N.S., Sell A.M., Visentainer L., Quirino Alves de Lima N., Zacarias J.M.V., Couceiro P., Shinzato A.H., Rosa M.S. (2022). Genetic Polymorphisms of Toll-like Receptors in Leprosy Patients from Southern Brazil. Front. Genet..

[88] Kang S.S., Abdel-Massih R., Brown R.A., Dierkhising R.A., Kremers W.K., Razonable R.R. (2012). Homozygosity for the Toll-like Receptor 2 R753Q Single-Nucleotide Polymorphism Is a Risk Factor for Cytomegalovirus Disease After Liver Transplantation. J. Infect. Dis..

[89] Chen L., Zheng L., Chen P., Liang G. (2020). Myeloid Differentiation Primary Response Protein 88 (MyD88): The Central Hub of TLR/IL-1R Signaling. J. Med. Chem..

[90] Saleh M.A., Ramadan M.M., Arram E.O. (2017). Toll-like Receptor-2 Arg753Gln and Arg677Trp Polymorphisms and Susceptibility to Pulmonary and Peritoneal Tuberculosis. Apmis.

[91] Pabst S., Yenice V., Lennarz M., Tuleta I., Nickenig G., Gillissen A., Grohé C. (2009). Toll-like Receptor 2 Gene Polymorphisms Arg677Trp and Arg753Gln in Chronic Obstructive Pulmonary Disease. Lung.

[92] Lee S.-H., Nishino M., Mazumdar T., Garcia G.E., Galfione M., Lee F.L., Lee C.L., Liang A.K., Kim J., Feng L. (2005). 16-kDa Prolactin Down-Regulates Inducible Nitric Oxide Synthase Expression Through Inhibition of the Signal Transducer and Activator of Transcription 1/Ifn Regulatory Factor-1 Pathway. Cancer Res..

[93] Liu S., Jia H., Hou S., Xin T., Guo X., Zhang G., Gao X., Li M., Zhu W., Zhu H. (2018). Recombinant Mtb9.8 of Mycobacterium Bovis Stimulates TNF-α and IL-1β Secretion by RAW264.7 Macrophages Through Activation of NF-κB Pathway via TLR2. Sci. Rep..

[94] Cai Y., Peng Y.B., Tang Z., Guo X.-L., Qing Y., Liang S., Jiang H., Dang W.-T., Ma Q., He C. (2014). Association of Toll-like Receptor 2 Polymorphisms with Gout. Biomed. Rep..

[95] Myles A., Aggarwal A. (2012). Lack of Association of Single Nucleotide Polymorphisms in Toll-like Receptors 2 and 4 with Enthesitis-Related Arthritis Category of Juvenile Idiopathic Arthritis in Indian Population. Rheumatol. Int..

[96] Semple C., Choi K.-Y.G., Kroeker A., Denechezhe L., Orr P., Mookherjee N., Larcombe L. (2019). Polymorphisms in the P2X7 Receptor, and Differential Expression of Toll-like Receptor-Mediated Cytokines and Defensins, in a Canadian Indigenous Group. Sci. Rep..

[97] Liakath F.B., Varatharajan S., Premkumar P.S., Syed C., Ward H., Kang G., Ajjampur S.S.R. (2021). Toll-like Receptors and Mannose Binding Lectin Gene Polymorphisms Associated With Cryptosporidial Diarrhea in Children in Southern India. Am. J. Trop. Med. Hyg..

[98] Woehrle T., Du W., Goetz A., Hsu H.Y., Joos T.O., Weiss M., Bauer U., Brueckner U.B., Marion Schneider E. (2008). Pathogen specific cytokine release reveals an effect of TLR2 Arg753Gln during Candida sepsis in humans. Cytokine.

[99] Khaled B.M., Noha A.S.M., Manal A.A.M., Engy S.M. (2020). Role of Toll-like Receptors 2 and 4 Genes Polymorphisms in Neonatal Sepsis in a Developing Country: A Pilot Study. J. Pediatr. Infect. Dis..

[100] Rosentul D.C., Delsing C.E., Jaeger M., Plantinga T.S., Oosting M., Costantini I., Venselaar H., Joosten L.A.B., van der Meer J.W., Dupont B. (2014). Gene polymorphisms in pattern recognition receptors and susceptibility to idiopathic recurrent vulvovaginal candidiasis. Front. Microbiol..

[101] Netea M.G., Brown G.D., Kullberg B.J., Gow N.A. (2008). An integrated model of the recognition of *Candida albicans* by the innate immune system. Nat. Rev. Microbiol..

[102] Plantinga T.S., Johnson M.D., Scott W.K., van de Vosse E., Velez Edwards D.R., Smith P.B., Alexander B.D., Yang J.C., Kremer D., Laird G.M. (2012). Toll-like receptor 1 polymorphisms increase susceptibility to candidemia. J. Infect. Dis..

[103] Carvalho A., Pasqualotto A.C., Pitzurra L., Romani L., Denning D.W., Rodrigues F. (2008). Polymorphisms in toll-like receptor genes and susceptibility to pulmonary aspergillosis. J. Infect. Dis..

[104] Hawn T.R., Verbon A., Lettinga K.D., Zhao L.P., Li S.S., Laws R.J., Skerrett S.J., Beutler B., Schroeder L., Nachman A. (2003). A common dominant TLR5 stop codon polymorphism abolishes flagellin signaling and is associated with susceptibility to legionnaires’ disease. J. Exp. Med..

[105] Carvalho A., De Luca A., Bozza S., Cunha C., D’Angelo C., Moretti S., Perruccio K., Iannitti R.G., Fallarino F., Pierini A. (2012). TLR3 essentially promotes protective class I-restricted memory CD8(+) T-cell responses to Aspergillus fumigatus in hematopoietic transplanted patients. Blood.

[106] Nahum A., Dadi H., Bates A., Roifman C.M. (2011). The L412F variant of Toll-like receptor 3 (TLR3) is associated with cutaneous candidiasis, increased susceptibility to cytomegalovirus, and autoimmunity. J. Allergy Clin. Immunol..

[107] Hatinguais R., Willment J.A., Brown G.D. (2022). C-type Lectin Receptors in Antifungal Immunity: Current Knowledge and Future Developments. Parasite Immunol..

[108] Goyal S., Castrillón-Betancur J.C., Klaile E., Slevogt H. (2018). The Interaction of Human Pathogenic Fungi with C-Type Lectin Receptors. Front. Immunol..

[109] Heine H., Zamyatina A. (2022). Therapeutic Targeting of TLR4 for Inflammation, Infection, and Cancer: A Perspective for Disaccharide Lipid A Mimetics. Pharmaceuticals.

[110] Miyauchi M., Murata M., Fukushima A., Sato T., Nakagawa M., Fujii T., Koseki N., Chiba N., Kashiwazaki Y. (2012). Optimization of cell-wall skeleton derived from Mycobacterium bovis BCG Tokyo 172 (SMP-105) emulsion in delayed-type hypersensitivity and antitumor models. Drug Discov. Ther..

[111] Araujo N.M., Chanes G.B., da Cruz K.A.S., de Rubio I.G.S., Villa L.L., Tamura R.E., Morale M.G. (2025). The antitumor effect of tlr4 inhibition in head and neck cancer cell lines. Sci. Rep..

